# Nutritional Composition of Tonka Bean (*Dipteryx odorata*) and Its Application as an Elder-Friendly Food with Gelling Agent

**DOI:** 10.3390/gels8110704

**Published:** 2022-10-31

**Authors:** Dah-Sol Kim, Fumiko Iida

**Affiliations:** Department of Food and Nutrition, Japan Women’s University, Tokyo 112-8681, Japan

**Keywords:** Tonka bean, gelling agent, viscosity, elder-friendly food

## Abstract

(1) Background: The purpose of this study was to compare the nutritional characteristics of Tonka beans according to the cooking method and to prove the feasibility of application as an elder-friendly food. (2) Methods: After analyzing the nutritive components, antioxidant activity, and anti-diabetic activity of raw, boiled, and roasted Tonka beans, custards, to which roasted Tonka beans were added, were prepared using a gelling agent to meet the KS viscosity standards (≤1500 mPa.s). (3) Results: The cooking methods decreased the nutritive factors in Tonka beans. However, while boiling caused significant losses, roasting led to minor losses. However, because the elderly should avoid eating uncooked foods for safety reasons, semi-solid elder-friendly food was manufactured with roasted Tonka beans, which caused minor losses compared to boiling. The concentration of each gelling agent satisfying the KS viscosity was less than 0.745% of locust bean gum, 0.734% of κ-carrageenan, and 1.094% of agar. (4) Conclusions: Roasted Tonka beans are suitable for use as an elder-friendly food for the health and safety of the elderly, and it will be possible to promote balanced food intake through the use of gelling agents for the elderly who have difficulty swallowing.

## 1. Introduction

*Dipteryx odorata* (commonly known as “Cumaru”, “Kumaru”, or “Brazilian teak”) is a species of flowering tree in the pea family, Fabaceae [1]. The tree is native to Central America and northern South America and is semi-deciduous. Its seeds are known as Tonka beans (sometimes “Tonkin beans” or “Tonquin beans”). They are black and wrinkled and have a smooth, brown interior. They normally contain about 1 to 3% of coumarin, which is responsible for the pleasant odor of the seeds [2]. As such, Tonka beans have a strong flavor similar to sweet woodruff due to its high content of coumarin. The flavor has been described as a complex mix of vanilla, almond, clove, cinnamon, and amaretto. However, coumarin is bitter in taste and can cause hemorrhages, liver damage, or heart attack with large doses. Therefore, it is a food additive controlled by many governments. However, because of that fact, there is a lot of misinformation about the toxicity of Tonka beans [1]. The reality is that at least 30 portions of Tonka beans are needed for coumarin levels to become dangerous. In France, for example, Tonka beans are often used in cooking (especially in desserts and stews). Additionally, Tonka beans have many industrial uses, such as medicinal properties [3]. People take Tonka beans in their daily lives to cure spasms, nausea, coughing, convulsion, tuberculosis, swelling due to lymphatic obstruction (lymphedema), and a parasitic disease called schistosomiasis [4]. Tonka bean can also help remove bacteria from our body naturally and clean up our biological systems from germs. In other words, when the flavor component of Tonka beans is consumed, secretion in the body increases, and bacteria is released out of the body through the mucous of the nose. It will also help remove mucous in the passage of the lung and detoxify, helping to clear them through coughing, and will also relieve asthma [2]. Despite its potential, Tonka beans are poorly adopted in agricultural systems due to the presence of toxic compounds. Therefore, in this study, in order to reveal the potential physiological benefits of whether Tonka bean can be used as an elder-friendly food material, the Tonka bean was selected as a research material.

Meanwhile, in order to effectively utilize beans as an elder-friendly food, it is necessary to inactivate anti-nutritional factors or to improve nutritional quality by adopting economically viable processing technique [5]. In addition, the elderly have a high risk of food poisoning due to reduced immunity due to aging, so they should eat processed foods such as heat treatment rather than raw food for food hygiene safety. Physical and biochemical ways applied to process beans include cooking, irradiation, enzyme treatment, fermentation, etc. [6]. Therefore, this study sought to find appropriate cooking methods to increase the significant nutritional properties of Tonka beans and not just to reveal the physiological benefits of Tonka beans. Furthermore, it was intended to develop viscoelastic custard as an elder-friendly food using Tonka beans cooked with an appropriate cooking method. This is because the demand for elder-friendly food continues to increase [7]. In particular, South Korea entered an aged society with a population ratio of more than 14% of the total population aged 65 or older in 2018, and as it is expected to enter an ultra-aged society in 2026, elder-friendly industries became very important. Therefore, Korea’s Ministry of Food and Rural Affairs quantified the actual hardness of chewable food for the elderly and recommended that elder-friendly foods be developed based on this information [8]. This was defined as Korean Industrial Standards (KS), and for solid food, the first stage was classified as a level that can be chewed with teeth, the second stage was classified as a level that can be chewed with the gums, and the third stage was classified as a level that can be chewed with the tongue. In addition, viscosity was quantified for liquid foods in consideration of not only difficulty in chewing but also difficulty in swallowing. In other words, since muscle weakness due to aging affects not only the mouth but also the esophagus, there may be a risk of choking when drinking beverages such as water without viscosity [7]. In addition, choking can lead to acute pneumonia and deaths in severe cases. However, drinking a slightly viscous drink can prevent this risk. Therefore, KS presented not only hardness for solid food but also appropriate viscosity criteria (≤1500 mPa.s) for liquid food. The government has been trying to expand various elder-friendly food industries by establishing these standards [6]. Therefore, in this study, it was attempted to determine whether Tonka bean can be used as an elder-friendly food and to develop a semi-solid type of elder-friendly food by using various gelling agents in custard added with Tonka beans.

The gelling agents used for that purpose were locust bean gum, κ-carrageenan, and agar. Locust bean gum is a galactomannan vegetable gum extracted from the seeds of a carob tree and is used as a gelling/thickening agent in food technology. This carob powder is produced from fruit pod after seed removal, while the gum is produced from seeds themselves [9]. Locust bean gum consists mainly of high-molecular-weight hydrocolloidal polysaccharides, which consists of galactose and mannose units bound by glycosidic bonds, which can be chemically described as galactomannan [10]. κ-Carrageenan is a family of natural linear sulfated polysaccharides extracted from red edible seaweed [11]. It is a large, highly flexible molecule that forms a curling spiral structure that can form various different gels at room temperature. Therefore, it is widely used in the food industry for gelling, thickening, and stabilizing. Additionally, it can be used to replace gelatin in confectionery and other food because it is an alternative to gelatin for vegetarians and vegans [12]. Agar is a jelly-like substance composed of polysaccharides obtained from the cell wall of red algae. It is used as a dessert ingredient throughout Asia and is also used as a solid substrate containing culture media for microbiological work. In addition, agar contains about 80% of dietary fiber, so it can act as an intestinal regulator and is also a popular weight-control diet in Asia. Once ingested, agar triples in size and absorbs water, as it makes consumers feel more full.

In summary, in this study, after establishing the optimal cooking method through a comparative analysis of physiological activity of Tonka beans according to the cooking method, we attempted to determine whether Tonka beans can be used as an elder-friendly food and to develop a viscoelastic elder-friendly food by using various gelling agents in custards added with Tonka beans cooked with an appropriate cooking method. In other words, it was intended to present research results on the design of special foods for the elderly from a recent industrial point of view, concentrating on viscosity, using Tonka beans and several thickeners.

## 2. Results and Discussion

### 2.1. Comparison of Fatty Acid Composition in Tonka Bean by Cooking Methods

The FA composition of raw, roasted, and boiled Tonka beans is compared in Table 1. In the case of raw Tonka bean, the mean content of unsaturated FAs among total body fat (38.909 g/100 g) was 34.695 g/100 g, which was about eight times higher than the mean content of saturated FAs (4.182 g/100 g). Among them, linoleic acid (C_18:2_) was the most abundant FA, with a mean content of 28.517 g/100 g, followed by an oleic acid content (4.863 g/100 g). Meanwhile, other FAs found showed similar contents between 0.005 and 1.150 g/100 g. Linoleic acid is a polyunsaturated ω-6 FA, which is one of the essential FAs for humans to consume through their diet. The intake of linoleic acid is known to be associated with lowering the risk of cardiovascular disease and premature death [13]. According to the preceding Meta-analyses of prospective cohort studies and randomized controlled trials suggest, high intake of linoleic acid, which replaces saturated FAs or carbohydrates, suggests that it has an appropriate advantages in preventing coronary artery disease. In addition, it has been reported that the intake of linoleic acid, a ω–6 unsaturated FA that constitutes >85~90% of dietary unsaturated FA intake, is very important for critical public health. Therefore, the current World Health Organization (WHO) dietary guidelines recommend increasing unsaturated FA intake instead of saturated FA intake to prevent cardiovascular disease and actively recommended that saturated FA intake does not exceed 10% of the total energy intake. Considering these, Tonka bean is likely to be a good choice as an alternative food that can increase unsaturated FA intake while limiting saturated FA intake. Similarly, in boiled and roasted Tonka beans, the mean content of linoleic acid was the most abundant at 26.322 and 27.447 g/100 g. Meanwhile, it was found that the FA composition of the Tonka bean changed according to the cooking method. Compared to raw Tonka bean, saturated and unsaturated FA contents were significantly low in roasted and boiled Tonka bean (*p* < 0.01). Specifically, among the saturated FAs, the contents of caprylic acid (C_8:0_), capric acid (C_10:0_), undecanoic acid (C_11:0_), lauric acid (C_12:0_), and myristic acid (C_14:0_) were significantly the highest in the raw Tonka bean, while the contents of tridecanoic acid (C_13:0_), henicosanoic acid (C_21:0_), behenic acid (C_22:0_), and lignoceric acid (C_24:0_) were the highest in the roasted Tonka bean (*p* < 0.05). In the case of other saturated FAs, there was no significant difference in the content according to the cooking method. Among the unsaturated FAs, the contents of oleic acid (C_18:1_), linoleic acid (C_18:2_), γ-linolenic acid (C_18:3n6_), icosenoic acid (C_20:1_), and erucic acid (C_22:1_) were significantly the highest in the raw Tonka bean, while the contents of α-linolenic acid (C_18:3n3_), eicosapentaenoic acid (C_20:5n3_), and docosahexaenoic acid (C_22:6n3_) were the highest in the roasted Tonka bean (*p* < 0.01). As such, roasting and boiling caused a significant loss in unsaturated FAs (*p* < 0.01).

On the other hand, roasted Tonka beans had a higher ω-3 (0.052 g/100 g) FA content compared to raw (0.042 g/100 g) and boiled Tonka beans (0.038 g/100 g). ω-3 FA helps prevent heart disease and stroke; helps relieve lupus, eczema, and rheumatoid arthritis; and serves as a protective role in cancer and other diseases [14]. Additionally, there was no significant difference in the ω-6 FA content between the cooking methods. ω-6 FA plays an important role in immune function, nervous system and cardiovascular function (especially reducing the risk of coronary heart disease and lowering excessive blood cholesterol levels), and the integrity of the epidermis. However, a diet with too high of a ω-6 FA content can increase inflammation, bringing a higher risk of many chronic diseases. A lower ratio of ω-6/ω-3 FAs is preferable to reduce the risk of many chronic diseases worldwide [15]. In this study, the ratio of ω-6/ω-3 FAs of roasted Tonka beans (13.741) was lower than that of raw (16.231) and boiled (17.201) Tonka beans. With Korea’s recent entry into a super aging society and the elderly’s interest in foods containing a large amount of omega-based FAs is increasing, the development of food for the elderly using roasted Tonka beans is expected to be used as the basis for the steadily growing elder-friendly food industry.

### 2.2. Comparison of Mineral Composition in Tonka Bean by Cooking Method

The mineral compositions of raw, roasted, and boiled Tonka beans are compared in Table 2. In the case of raw Tonka beans, the mean content of Fe (198.583 g/100 g) was the highest, followed by Mn, K, Ca, Na, Zn, P, Mg, and Cu. Fe is a mineral that the body needs to grow and develop [16]. The human body uses Fe to make hemoglobin, a protein in red blood cells that carries oxygen from the lungs to all parts of the body, and myoglobin, a protein that supplies oxygen to the muscles. In terms of roasted (208.959 g/100 g) and boiled (187.022 g/100 g) Tonka beans, the mean content of Fe tended to be the highest. Mo and Se were not detected. Meanwhile, it was found that the mineral composition of the Tonka bean changed according to the cooking method. Compared to raw Tonka beans, Na, Ca, Fe, Zn, and Mn contents were significantly low in roasted and boiled Tonka beans, while K and P contents were the highest in roasted Tonka beans. Additionally, there was no significant difference in the mean content of Mg and Cu according to the cooking method.

Cooking food helps digestion and increases the absorption of many nutrients into the body. For example, the protein of cooked eggs is 180% better digested in the body than the protein of raw eggs [17]. However, some cooking methods also reduce several major nutrients such as water-soluble vitamins, fat-soluble vitamins, and minerals (primarily K, Mg, Na, and Ca). In particular, it has been reported that boiling reduces nutrient content compared to any other cooking method [18]. Minerals can be leached from food when immersed in hot water because they are water-soluble. According to Kimura and Itokawa (1990)’s [19] previous study, mineral loss of mineral was the highest in the process of squeezing after boiling among various cooking methods, followed by soaking, parching, frying, and stewing. In addition, minerals are sensitive to temperature and can change with heat treatment. Of course, raw foods generally retain more minerals than cooked ones. However, all foods must be cooked for safety’s sake of the elderly. Light cooking can also boost the absorption of certain nutrients and healthful plant chemicals. Therefore, in order to use Tonka beans as an elder-friendly food material, it is considered appropriate to roast.

### 2.3. Comparison of Antioxidant Effects of Tonka Bean by Cooking Method

#### 2.3.1. Total Polyphenol Content

The antioxidant effects of raw, roasted, and boiled Tonka bean are compared in Table 3. Boiling resulted in a significant loss of polyphenols, while roasting resulted in an apparent increase in polyphenols. It was similar to the results of previous studies by Asfaw and Tefera (2020) [20]. According to previous studies, it has been reported that the loss of polyphenols may be due to leaching of water-soluble compounds into cooking water as well as decomposition during boiling. Alternatively, it may be because the phenol compound is partially decomposed and/or bonded to the polymer structure when boiled. On the other hand, it has been reported that roasting contributes to the improvement of phenol content and its total antioxidant activity due to the release of combined phenol compounds and the formation of novel compounds with antioxidant activity [21].

#### 2.3.2. Total Flavonoid Content

The mean content of the total flavonoid increased after roasting compared to raw Tonka bean, while decreased after boiling. The loss of flavonoids after boiling may be due to the fact that flavonoid compounds are leached into cooking water and pyrolyzed for complex reasons [22]. Therefore, dry-heat cooking has been proposed as a preferred cooking method over moist-heat cooking for foods, due to high consumer acceptability, low nutrient loss, and high availability of phenolic compounds. In particular, due to the antioxidant ability of phenolic compounds including flavonoids and phenolic acids, interest in the prevention or treatment of cancer through food intake containing these components is increasing [23].

#### 2.3.3. DPPH Free Radical Scavenging Activity

DPPH free radical scavenging activity decreased after cooking. This change is because not only the breakdown of antioxidant compounds during boiling but also the leaching of water-soluble compounds into the cooking water [20]. Furthermore, heating generally causes acceleration of the initiation reaction, thereby reducing the activity of the antioxidant [24]. This is because temperature is one of the important factors influencing antioxidant activity. However, boiling resulted in significant loss of DPPH free radical scavenging activity, while roasting led to minor loss of the DPPH free radical scavenging activity. In other words, among the cooking conditions (roasting and boiling) used in this study, roasting was found to be a better way to conserve DPPH free radical scavenging activity than boiling. In order to prevent the harmful role of free radicals that cause various diseases including cancer, such free radical scavenging activities are very important [25].

#### 2.3.4. ABTS Free Radical Scavenging Activity

ABTS free radical scavenging activity decreased after cooking. This may be due to the fact that antioxidant compounds are leached into cooking water and pyrolyzed, such as the DPPH free radical scavenging activity discussed above [22]. However, boiling resulted in significant loss of ABTS free radical scavenging activity, while roasting led to minor loss of the ABTS free radical scavenging activity. Meanwhile, free radicals, such as ABTS, are molecules produced when our bodies are exposed to tobacco smoke or radiation [26]. However, eliminating this ABTS can protect body from heart disease, cancer, and other diseases by protecting cells. Therefore, it is important to know the antioxidant efficacy of food to protect it from oxidative damage to prevent harmful changes and loss of commercial and nutritional value.

#### 2.3.5. Superoxide Dimutase Activity

Superoxide dismutase activity decreased after cooking. However, while boiling resulted in significant loss of SOD activity, roasting resulted in minor loss of the SOD activity. These enzymes serve as good treatments for reactive oxygen species-mediated diseases [27]. In particular, SOD plays a very important defense against oxidative stress in the body. The SOD families activity depends on a specific redox active metal ion and may be manganese, iron, copper, or nickel ions depending on the SOD molecule [28]. According to the results of this study, Mn and Fe contents were found to be the highest in Tonka bean, which is thought to have an effect on SOD activity.

#### 2.3.6. Ferric Reducing Antioxidant Power

Ferric reducing antioxidant power decreased after cooking. However, boiling resulted in significant loss of ferric reducing antioxidant power, while roasting led to minor loss of the ferric reducing antioxidant power. The higher the intake of antioxidant-rich fruits, vegetables, and legumes, the lower the risk of chronic oxidative stress-related diseases such as cardiovascular disease. Therefore, among the cooking methods used in this study, roasting was found to be a better way to conserve ferric, which reduces antioxidant power than boiling. Meanwhile, maintenance factors of the antioxidants in other foods may vary depending on the different food matrix, compound profile, and specific cooking methods and conditions [21]. Therefore, the results of this study cannot be simply be applied to other foods containing similar or even the same compounds. Cooking effects on antioxidants in different types of foods should be investigated and evaluated on a case-by-case basis.

### 2.4. Comparison of Antidiabetic Effects of Tonka Bean by Cooking Method

#### 2.4.1. α-Glucosidase Inhibition

Antidiabetic effects of raw, roasted, and boiled Tonka bean is compared in Table 3. Raw Tonka beans showed the most effective inhibitory effect on α-glucosidase activity (67.358%) compared to the cooked sample. However, among the cooking methods of this study, roasting (63.138%) led to a minor loss of α-glucosidase inhibition rather than boiling. α-glucosidase is one of the carbohydrate digestive enzymes found in the brush-border surface membrane of intestinal cells [29]. This hydrolyzes disaccharides and oligosaccharides present in the intestine (lumen), promoting the production of glucose for intestinal absorption. Therefore, the inhibition of α-glucosidase, a carbohydrate digestive enzyme, is known as one of the most important approaches to managing obesity and diabetes.

#### 2.4.2. α-Amylase Inhibition

Raw Tonka beans showed the most effective inhibitory effect on α-amylase activity (60.651%) compared to cooked samples. Additionally, there was no significant difference in α-amylase inhibition between cooking methods. This is because the lower the temperature, the more inactivated the enzyme’s reaction, while the higher the optimal temperature, the more denatured the enzyme. It is believed that this is because the enzyme is destroyed, which reduces the reaction, and eventually stops the enzyme [30].

### 2.5. Viscosity of Custard Added with Roasted Tonka Bean by Gelling Agent

Cooked custard is a weak gel, is viscous, and is thixotropic; unlike many other thixotropic liquids, it does not recover lost viscosity over time, while the more manipulated it is, the easier it is to stir [31]. Considering these characteristics, a custard as a semi-solid type of elder-friendly food was developed by adding a natural gelling agent. The gelling agents used for this purpose were locust bean gum, κ-carrageenan, and agar, which are widely used commercially in the food industry, for their gelling, thickening, and stabilizing properties. They were selected as the subject of this study because they had characteristics such as not damaging the unique taste of food. Table 4 shows the viscosity results of the custard added with roasted Tonka beans according to the concentration of each gelling agent.

**Figure 1:**
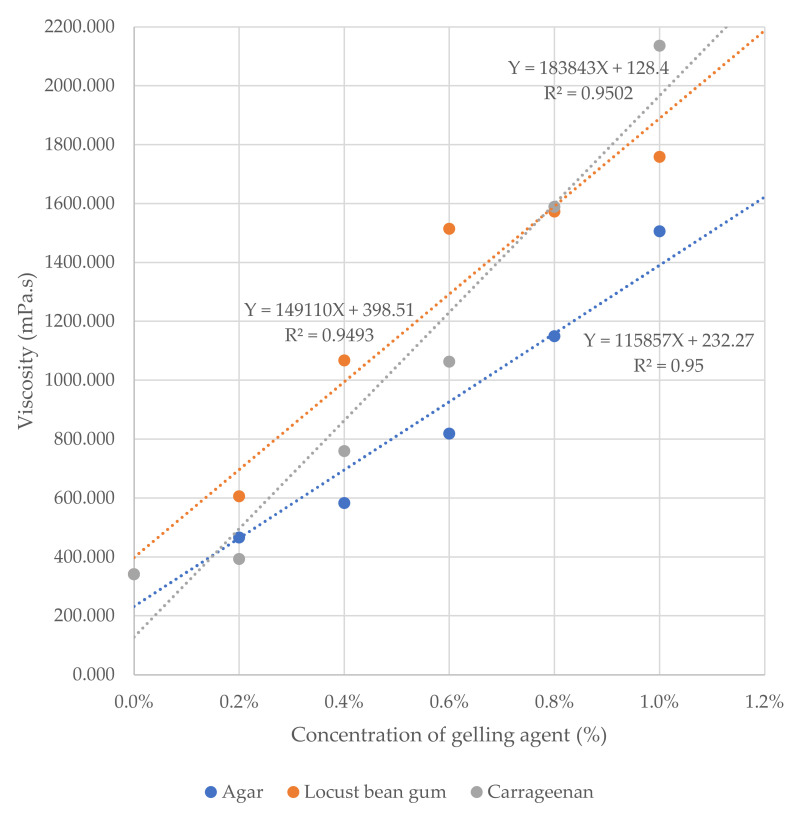
Regression equation for the viscosity of the custard added with roasted Tonka bean by gelling agent. The Y-scale is “viscosity (mPa.s)”, and the X-scale is “concentration of gelling agent (%)”. The coefficient of determination (R^2^) is a number between 0 and 1 that measures how well a statistical model predicts an outcome, and a higher coefficient is an indicator of a better goodness of fit for the observations. KS for elder-friendly foods has set a standard that a viscosity of 1500 mPa.s or less is appropriate for fluid foods for the elderly.

As a result of adding to the custard by concentration in consideration of the locust bean gum, the viscosity significantly (*p* < 0.001) increased as the concentration increased. Although it may be a natural result, in order to predict the concentration of locust bean gum suitable for KS viscosity level (≤1500 mPa.s), a regression equation was derived based on these results. Accordingly, the linear regression equation was derived as “Y = 149,110X + 398.51” with high accuracy (R^2^ = 0.9493), and the concentration of the locust bean gum suitable for KS viscosity level was 0.745% or less (Figure 1). As a result of adding to the custard by concentration in consideration of the κ-carrageenan, the viscosity significantly (*p* < 0.001) increased as the concentration increased. This may also be a natural result, but based on this result, the linear regression equation was derived as “Y = 183,843X + 128.40” with high accuracy (R^2^ = 0.9502), and the concentration of κ-carrageenan suitable for KS viscosity level was 0.734% or less. As a result of adding to the custard by concentration in consideration of the agar, the viscosity significantly (*p* < 0.001) increased as the concentration increased. This may also be a natural result, but based on this result, the linear regression equation was derived as “Y = 115,857X + 232.27” with high accuracy (R^2^ = 0.95), and the concentration of agar suitable for KS viscosity level was 1.094% or less.

According to previous studies, considering the KS viscosity, it was found that lingon berry juice, which was added less than 0.384% of locust bean gum, 0.341% of guar gum, and 0.596% of xanthan gum, had an appropriate viscosity for the elderly to consume [32]. These differences are believed to be due to the acidity affecting the viscosity. At higher pH values, the polymer was believed to act as an acid and, at lower values, as a base, with the repulsion between charges on oxygen atoms then increasing the chain length and, hence, the viscosity [33]. According to a previous study, an increase in viscosity has been observed by decreasing the pH. High-viscosity dispersions with pseudoplastic and thixotropic flow behaviors were formed at pH 7.0 and 7.5, whereas weak gels were obtained at pH 6.0 and 6.5 [34]. According to another previous study, the acids caused reduction in gelatinization temperature and enthalpy of gelatinization of both starches. The starch gels became softer, less cohesive, elastic, and gummy when acids were added. These changes may indicate the degradation of the starch molecules by the acids [35]. In addition, it is believed that the temperature, composition, and condition of the sample, additives, etc. may have affected the viscosity.

Comparing these three gelling agents, it was found that there was a significant (*p* < 0.01) difference between the three gelling agents at all concentrations. Additionally, while the viscosity tended to increase gradually as the concentration of agar increased, the viscosity increased exponentially as the concentration of the κ-carrageenan increased. In other words, it means that the κ-carrageenan can make a significant change in viscosity even with a smaller amount than other gelling agents. Considering these characteristics, it can be said that κ-carrageenan has an economic advantage in the field of the elder-friendly food industry.

## 3. Conclusions

This study showed that different cooking methods can significantly affect nutritional components, antioxidant activities, and antidiabetic activities of Tonka beans. Cooking methods significantly decreased nutritive factors in Tonka beans. These findings would be useful for consumers when choosing best cooking methods. Meanwhile, retention factors for nutritional components in other foods are likely to be different due to the different food matrix, compound profile, and specific cooking methods and conditions. Therefore, the results from this study cannot be simply extrapolated to other foods that contain similar or even same components. Cooking effects on nutritive factors of different types of food should be investigated and evaluated on a case-by-case basis. Meanwhile, people over 65 years old are at higher risk of dying from foodborne diseases due to changes in organs and body systems as their bodies age [36]. Therefore, elderly people should avoid eating uncooked food for safety, and that is why we should cook Tonka beans. Therefore, in this study, roasting led to a minor loss than boiling, so semi-solid type elder-friendly food to which roasted Tonka bean is added was developed to meet the KS viscosity standard (≤1500 mPa.s) using a gelling agent. The concentration of each gelling agent satisfying the standard was 0.745% or less of locust bean gum, 0.734% or less of κ-carrageenan, and 1.094% or less of agar, and among them, the κ-carrageenan may have a large change in viscosity even in a smaller amount than other gelling agents. In other words, it can be said that κ-carrageenan has an economic advantage in the elder-friendly food industry.

## 4. Materials and Methods

### 4.1. Sample Preparation

In this study, Brazilian Tonka beans (MOSmall, Seoul, Korea) were used as a sample. Through preliminary experiments, it was found that roasting Tonka beans in a heated pan at 140 °C for 15 min or boiling it in water (1 L) for 10 min at 100 °C was the best condition to minimize the bitterness of Tonka beans. Additionally, in order to make a sample for this experiment, a broken Tonka bean was first screened visually, washed lightly with running water, dried with a kitchen towel, and divided into three portions (100 g each). The first portion was raw and used for analysis. The second portion was roasted in a heated pan at 140 °C for 15 min to remove the bitterness of the Tonka bean. The third portion was boiled with water (1 L) at 100 °C for 10 min to remove the bitterness of the Tonka bean. Each sample was freeze-dried with a freeze dryer (DC401, Yamato Scientific Co., Ltd., Tokyo, Japan) for 72 h, then powdered with a grinder (HL7756/00, Philips Ltd., Sallewal, India), and used in a physicochemical experiment.

### 4.2. Analysis of Fatty Acid Composition

The identification and quantitation of fatty acids (FAs) were performed by gas chromatography (GC) using an Agilent 7890 series GC (Agilent technologies 7890-A, Palo Alto, CA, USA) systemors [8]. Each sample (20 mg) was saponified with 0.5 N methanolic NaOH (3 mL) at 85 °C for 10 min and cooled to room temperature. Then, 14% BF_3_ in methanol (3 mL) was added and methylated with at 85 °C for 10 min to convert FAs into methyl esters. This was re-cooled to room temperature, and then, isooctane (3 mL) and saturated NaCl solution (5 mL) were added and vortexed. After that, an upper isooctane layer containing FA methyl ester was taken, passed through anhydrous Na_2_SO_4_ column, and 1 μL of it was injected at a split ratio of 200:1 into a GC equipped with a flame ionization detector and a fused silica capillary column (100 m × 0.25 mm i.d. × 0.2 μm film thickness, Supelco SP-2560, Bellefonte, PA, USA). Helium was used as the carrier gas at a flow rate of 1 mL min^−1^. The temperatures of the injector and detector were maintained at 225 and 285 °C, respectively. The temperature of the oven was initially maintained at 100 °C for 4 min, then raised to 240 °C at a speed of 3 °C min^−1^, and then maintained at 240 °C for 17 min. FA methyl esters were identified by comparing the reference standard (Supelco 37 component FA methyl esters mix, Bellefonte, PA, USA) and retention time.

### 4.3. Analysis of Mineral Composition

The mineral composition of each sample was analyzed using the method described by Kim and Joo (2019) [8]. Each sample (20 mg) was ashed at 550 °C in the oven, boiled with 10% HCl (10 mL), and filtered; 0.7 g was taken, placed in a Teflon digestion flask, and nitric acid (10 mL), and 30% H_2_O_2_ (3 mL) were added. This was digested in two steps using 1000 W power at a maximum temperature of 200 °C. Thereafter, it was cooled at room temperature and transferred to a 25 mL volumetric flask to fill the remaining volume with a 5% HCl solution. This was quantified into an inductively coupled plasma optical emission spectrometer (ICP-OES; Vista MPX, Varian, Mulgrave, VIC, Australia) with radio frequency source of 40 MHz, charge coupled devices simultaneous solid-state detector, peristaltic pump, seaspray nebulizer connected to cyclonic spray chamber, and 99.996% high-purity argon (Ar; air liquid). The system was controlled by the ICP Expert Software: 1000 W of forward power, 1.5 L min^−1^ of auxiliary argon (Ar) flow, 0.9 L min−1 of nebulizer Ar flow, 15 L min−1 as the cooling Ar flow rate, 2 points of background correction, 10 s of integration and reading time, and 3 as the replication number. The analysis wavelengths were 317.9 nm of calcium (Ca), 324.8 nm of copper (Cu), 234.4 nm of iron (Fe), 766.5 nm of potassium (K), 280.3 nm of magnesium (Mg), 260.6 nm of manganese (Mn), 202 nm of molybdenum (Mo), 589 nm of sodium (Na), 213.6 nm of phosphorus (P), 196 nm of selenium (Se), and 206.2 nm of zinc (Zn).

### 4.4. Analysis of Antioxidant Effects

#### 4.4.1. Sample Preparation

Approximately 5 g of samples was extracted with 70% ethanol for 18 h. All extracts were filtered using Whatman No. 5 filters (Tokyo, Japan). Each filtrate (40 μL) was used as a sample for physiological experiments.

#### 4.4.2. Total Polyphenol Content

Each filtrate (40 μL) was transferred to a 10 mL screw cap tube, and then, a 10-fold diluted folin-ciocalteau reagent (800 μL) was added, mixed well, and left in a dark place for 5 min [37]. A 7% sodium carbonate aqueous solution (800 μL) was added thereto; the remaining volume was filled with distilled water (DW; 360 μL), mixed well, and left in a dark place at room temperature for 2 h. Its absorbance was measured at 760 nm using a UV visible spectrophotometer (T60UV, PG instruments Ltd., Lutterworth, UK). The polyphenol content was expressed as μg of gallic acid equivalent per g of the sample weight.

#### 4.4.3. Total Flavonoid Content

Each filtrate (0.5 mL) was mixed with 95% ethanol (1.5 mL), 10% aluminum chloride hexahydrate (0.1 mL), 1 M potassium acetate (0.1 mL), and DW (2.8 mL) and left at room temperature for 40 min in a dark place [37]. Its absorbance was measured at 415 nm with a UV visible spectrophotometer (T60UV, PG instruments Ltd., Lutterworth, UK). The flavonoid content was expressed in μg of rutin equivalent per g of sample weight.

#### 4.4.4. DPPH Radical Scavenging Activity

Each filtrate (50 µL) was placed in a cuvette, and 6 × 10^−5^ M ethanolic solution (2 mL) of 2,20-diphenyl-1-picrylhydrazyl (DPPH) was added [32]. This was left in a dark place at room temperature for 1 h, and then, the reduced absorbance at 515 nm was measured with a UV visible spectrophotometer (T60UV, PG instruments Ltd., Lutterworth, UK). In addition, DPPH radical without antioxidant was set as a control group, and its absorbance was measured to calculate percentage inhibition for the experimental group. DPPH radical scavenging activity was expressed in μmol of trolox equivalent per g of sample weight.

#### 4.4.5. ABTS Radical Scavenging Activity

The reaction mixture (1 mL) contained the filtrate (10 µL), myoglobin solution (20 µL), and ABTS reagent (150 µL; 10 mL ABTS and 25 µL 3% H_2_O_2_) [32]. This was left at room temperature for 10 min in a dark place, and then, absorbance was measured at 405 nm using a UV visible spectrophotometer (T60UV, PG instruments Ltd., Lutterworth, UK). In addition, ABTS radical without antioxidant was set as a control group, and its absorbance was measured to calculate the inhibition rate for the experimental group. ABTS radical scavenging activity was expressed in μmol of trolox equivalent per g of sample weight.

#### 4.4.6. Superoxide Dimutase Activity

Superoxide dismutase (SOD) activity was measured by a nitro blue tetrazolium (NBT) photochemical analysis method of Talukdar and Talukdar (2013) [38]. Each filtrate (20 μL) and a solution (1 mL) containing a 50 mM K-phosphate buffer (pH 7.8), 9.9 mM L-methionine, 57 μM NBT, and 0.025% triton-X-100 were mixed. After adding a riboflavin solution (10 μL; 0.044 mg mL^−1^), absorbance was measured at 560 nm using UV visible spectrophotometer (T60UV, PG instruments Ltd., Lutterworth, UK). SOD activity was expressed in U of trolox equivalent per mg of sample weight.

#### 4.4.7. Ferric Reducing Antioxidant Power

Plasma reagents (300 µL) for measuring ferric reducing antioxidant power (FRAP) was prepared by mixing an acetate buffer (25 mL), 2,4,6-Tris(2-pyridyl)-s-triazine solution (2.5 mL), and ferric chloride solution (2.5 mL) [32]. After heating at 37 °C, each filtrate (10 µL) and DW (30 µL) were added and left in a dark place at room temperature for 4 min. Its absorbance was measured at 593 nm using a UV visible spectrophotometer (T60UV, PG instruments Ltd., Lutterworth, UK). FRAP was expressed in mg of trolox equivalent per g of sample weight.

### 4.5. Analysis of Antidiabetic Effects

#### 4.5.1. α-Glucosidase Inhibition

One hundred mg of intestinal acetone powder (Sigma-Aldrich, St. Louis, MO, USA) was dissolved in 1 mL of 0.1 M maleate buffer (pH 6) and homogenized for 6 min [39]. This was centrifuged at 3000× g for 30 min, a supernatant was taken, diluted with buffer solution at 1:2 (*v*/*v*), and used as an α-glucosidase enzyme solution. This enzyme solution (20 mL), each filtrate (20 mL), and 2% sucrose in maleate buffer (20 mL) were mixed and pre-incubated at 378 °C for 1 h. This was heated in a water bath at 100 °C for 10 min to stop the enzyme reaction, and then 20 mL was taken, mixed with a color reagent (3 mL; Glucose CII-Test Wako, Wako Pure Chemical Industries), and incubated at 378 °C for 5 min. Its absorbance was measured at 505 nm with UV visible spectrophotometer (T60UV, PG instruments Ltd., Lutterworth, UK). In addition, the control group replaced the sample solution with a buffer solution and proceeded in the same manner as described above, and the inhibition rate for the experimental group was calculated.

#### 4.5.2. α-Amylase Inhibition

Each filtrate (500 μL) was mixed with a 0.02 M sodium phosphate buffer (500 μL; pH 6.9 with 0.006 M sodium chloride) containing a 0.5 mg/mL porcine pancreatic α-amylase solution and pre-incubated at 25 °C for 10 min [37]. Thereafter, 1% starch solution (500 μL) in 0.02 M sodium phosphate buffer (pH 6.9 with 0.006 M sodium chloride) was added and incubated at 25 °C for 10 min, and then, 3,5-dinitrosalicylic acid color reagent (1 mL) was added to stop the enzyme reaction. This was immersed in a boiling water bath for 5 min, cooled to room temperature, and diluted with DW (10 mL). Its absorbance was measured at 540 nm using a UV visible spectrophotometer (T60UV, PG Instruments Ltd., Lutterworth, UK). In addition, the control group replaced the sample solution with a buffer solution and proceeded in the same manner as described above, and the inhibition rate for the experimental group was calculated.

### 4.6. Development of Custard with Tonka Bean by Gelling Agent for the Elderly

#### 4.6.1. Preparation Method

First, pour milk (300 mL) into a microwavable bowl, stir in granulated sugar (50 g) and gelling agent (0~1.0% of custard mixture weight; locust bean gum, κ-carrageenan, agar; 99.99% purity; ESfood Co., Gyeonggi-do, Korea), and microwave it for 2 min at 600 W. In a separate bowl, crack in 3 whole eggs, whisk, add heavy cream (100 mL), and whisk again. Now, add ground roasted Tonka bean (1 g) and stir again. Take the milk mixture from the microwave and add a little bit to the egg and cream mix, and mix to temper the eggs (if you pour everything in at once, the hot milk might make the eggs scramble and become lumpy). Add the rest of the hot milk mixture little by little and pour through a strainer to make sure there are no lumps. Then, slowly pour the custard mixture (≈595 g) into the mold, cover with plastic wrap, and put in the refrigerator for 30 min.

#### 4.6.2. Analysis of Viscosity

Viscosity was measured using a rotational viscometer (VR-3000, model-L) equipped with an L-3 spindle. The rotation speed was set to 50 rpm, the measurement time was set at to 60 s, and the reading was recorded as centipoise (mPa.s).

### 4.7. Statistical Analysis

The change in the nutritional composition of Tonka beans according to the cooking method was statistically analyzed to find the optimal cooking method. Furthermore, a statistical analysis was conducted to determine the effect of the type of gelling agent on the development of elder-friendly foods using Tonka beans pretreated with the optimal cooking method. The experimental results were tested using one-way analysis of variance (ANOVA). This test performed a Scheffe post hoc test to analyze significant differences between test groups when the results were significant. This statistical analysis was performed using the IBM SPSS statistics program (Version 23.0, GraphPad Software Inc., San Diego, CA, USA), and if the *p*-value was less than 0.05 (*p* < 0.05), it was judged to be statistically significant. Furthermore, in order to develop an elder-friendly food suitable for KS viscosity, a linear regression equation was obtained to derive the optimal gelling agent concentration.

## Figures and Tables

**Table 1:** Fatty acid composition of raw, roasted, and boiled Tonka beans.

		Cooking Method (g/100 g) ^1^			F-Value ^2^ (*p*-Value)
		Raw	Roasting	Boiling	
Saturated fatty acid	Caproic acid (C_6:0_)	0.005 ± 0.001	0.005 ± 0.000	0.005 ± 0.001	0.500 (0.630)
	Caprylic acid (C_8:0_)	0.523 ± 0.006 ^a^	0.506 ± 0.014 ^ab^	0.483 ± 0.013 ^b^	8.610 * (0.017)
	Capric acid (C_10:0_)	0.105 ± 0.002 ^a^	0.100 ± 0.003 ^ab^	0.095 ± 0.003 ^b^	8.044 * (0.020)
	Undecanoic acid (C_11:0_)	1.073 ± 0.022 ^a^	1.037 ± 0.051 ^ab^	0.967 ± 0.021 ^b^	7.571 * (0.023)
	Lauric acid (C_12:0_)	0.082 ± 0.001 ^a^	0.079 ± 0.002 ^ab^	0.077 ± 0.001 ^b^	10.167 * (0.012)
	Tridecanoic acid (C_13:0_)	0.026 ± 0.001 ^ab^	0.027 ± 0.000 ^a^	0.026 ± 0.001 ^b^	6.000 * (0.037)
	Myristic acid (C_14:0_)	1.150 ± 0.015 ^a^	1.124 ± 0.018 ^ab^	1.094 ± 0.021 ^b^	7.415 * (0.024)
	Pentadecylic acid (C_15:0_)	0.333 ± 0.003	0.339 ± 0.005	0.327 ± 0.006	4.937 (0.054)
	Heptadecanoic acid (C_17:0_)	0.589 ± 0.001	0.597 ± 0.011	0.582 ± 0.011	1.736 (0.254)
	Arachidic acid (C_20:0_)	0.150 ± 0.001	0.154 ± 0.004	0.148 ± 0.002	4.750 (0.058)
	Henicosanoic acid (C_21:0_)	0.015 ± 0.001 ^ab^	0.016 ± 0.000 ^a^	0.015 ± 0.000 ^b^	7.000 * (0.027)
	Behenic acid (C_22:0_)	0.090 ± 0.001 ^ab^	0.091 ± 0.001 ^a^	0.087 ± 0.002 ^b^	7.588 * (0.023)
	Tricosanoic acid (C_23:0_)	0.027 ± 0.000	0.027 ± 0.000	0.027 ± 0.000	1.500 (0.296)
	Lignoceric acid (C_24:0_)	0.012 ± 0.000 ^ab^	0.013 ± 0.000 ^a^	0.012 ± 0.000 ^b^	7.000 * (0.027)
	Total	4.182 ± 0.038 ^a^	4.115 ± 0.065 ^a^	3.944 ± 0.080 ^b^	11.176 ** (0.009)
Unsaturated fatty acid	Palmitoleic acid (C_16:1_)	0.510 ± 0.015	0.498 ± 0.001	0.492 ± 0.006	3.224 (0.112)
	Oleic acid (C_18:1_)	4.863 ± 0.116 ^a^	4.693 ± 0.043 ^ab^	4.566 ± 0.143 ^b^	5.568 * (0.043)
	Linoleic acid (C_18:2_)	28.517 ± 0.883 ^a^	27.447 ± 0.082 ^ab^	26.322 ± 0.075 ^b^	13.709 ** (0.006)
	α-Linolenic acid (C_18:3n3_)	0.025 ± 0.002 ^b^	0.031 ± 0.003 ^a^	0.024 ± 0.000 ^b^	10.939 * (0.010)
	γ-Linolenic acid (C_18:3n6_)	0.638 ± 0.009	0.631 ± 0.002	0.624 ± 0.011	2.342 (0.177)
	Icosenoic acid (C_20:1_)	0.083 ± 0.003 ^a^	0.080 ± 0.001 ^ab^	0.077 ± 0.000 ^b^	6.333 * (0.033)
	Eicosadienoic acid (C_20:2n6_)	0.036 ± 0.001	0.035 ± 0.002	0.033 ± 0.001	2.867 (0.134)
	Eicosapentaenoic acid (C_20:5n3_)	0.006 ± 0.001 ^ab^	0.008 ± 0.001 ^a^	0.005 ± 0.000 ^b^	7.000 * (0.027)
	Erucic acid (C_22:1_)	0.006 ± 0.000 ^a^	0.005 ± 0.001 ^ab^	0.004 ± 0.000 ^b^	9.000 * (0.016)
	Docosahexaenoic acid (C_22:6n3_)	0.011 ± 0.001 ^ab^	0.014 ± 0.002 ^a^	0.010 ± 0.000 ^b^	9.056 * (0.015)
	Total	34.695 ± 1.008	33.442 ± 0.125	32.157 ± 0.237	13.319 ** (0.006)
ω-3 Fatty acid		0.042 ± 0.003	0.052 ± 0.007	0.038 ± 0.001	
ω-6 Fatty acid		0.674 ± 0.010	0.667 ± 0.003	0.657 ± 0.012	
Total body fat		38.909 ± 1.074	37.526 ± 0.223	36.101 ± 0.317	2.832 (0.136)

^1^ Results are shown as mean value in triplicate. ^2^ One-way ANOVA was used, and different letters in the same row (a~b) imply significant differences at * *p* < 0.05 and ** *p* < 0.01, respectively.

**Table 2:** Mineral composition of raw, roasted, and boiled Tonka beans.

	Cooking Method (g/100 g) ^1^			F-Value ^2^ (*p*-Value)
	Raw	Roasting	Boiling	
Na	32.623 ± 1.409 ^a^	28.805 ± 1.423 ^b^	26.846 ± 1.311 ^b^	13.565 ** (0.006)
K	56.098 ± 2.002 ^ab^	58.904 ± 1.581 ^a^	52.795 ± 2.091 ^b^	7.735 * (0.022)
Ca	32.536 ± 0.844 ^a^	31.059 ± 1.020 ^ab^	29.377 ± 1.098 ^b^	7.602 * (0.023)
Mg	10.613 ± 0.172	10.616 ± 0.338	9.958 ± 0.495	3.323 (0.107)
P	15.935 ± 0.595 ^ab^	16.750 ± 0.458 ^a^	14.984 ± 0.554 ^b^	8.070 * (0.020)
Fe	208.959 ± 5.870 ^a^	198.583 ± 7.537 ^ab^	187.022 ± 6.188 ^b^	8.365 * (0.018)
Zn	20.547 ± 0.581 ^a^	19.457 ± 0.851 ^ab^	18.149 ± 0.668 ^b^	8.606 * (0.017)
Cu	5.301 ± 0.083	5.307 ± 0.153	5.002 ± 0.198	3.954 (0.080)
Mn	102.366 ± 2.898 ^a^	97.494 ± 3.341 ^ab^	92.754 ± 2.206 ^b^	8.511 * (0.018)
Mo	N.D. ^3^	N.D.	N.D.	
Se	N.D.	N.D.	N.D.	

^1^ The results were expressed as the mean value for the three repeated experiments. ^2^ One-way ANOVA was used, and different letters in the same row (a~b) imply significant differences at * *p* < 0.05 and ** *p* < 0.01, respectively. ^3^ N.D. means undetected.

**Table 3:** Physicochemical effects of raw, roasted, and boiled Tonka beans.

		Cooking Method ^1^			F-Value ^2^ (*p*-Value)
		Raw	Roasting	Boiling	
Antioxidant effect	Total polyphenol content (GAE μg/g)	4739.369 ± 230.863 ^ab^	5066.658 ± 108.545 ^a^	4462.524 ± 141.384 ^b^	9.676 * (0.013)
	Total flavonoid content (RU μg/g)	488.833 ± 15.926 ^ab^	514.111 ± 10.593 ^a^	473.000 ± 7.603 ^b^	9.134 * (0.015)
	DPPH free radical scavenging activity (Trolox μmol/g)	42.138 ± 1.981 ^a^	40.341 ± 2.037 ^ab^	36.907 ± 1.832 ^b^	5.563 * (0.043)
	ABTS free radical scavenging activity (Trolox μmol/g)	57.407 ± 0.175	57.199 ± 0.306	57.125 ± 0.094	1.442 (0.308)
	Superoxide dismutase activity (U/mg)	7.484 ± 0.288	7.286 ± 0.315	7.220 ± 0.266	0.671 (0.546)
	Ferric reducing antioxidant power (Trolox mg/g)	5.422 ± 0.216 ^a^	4.989 ± 0.245 ^ab^	4.737 ± 0.191 ^b^	7.546 * (0.023)
Antidiabetic effect	α-Glucosidase inhibition (%)	67.358 ± 3.237 ^a^	63.138 ± 2.485 ^ab^	59.038 ± 2.792 ^b^	6.370 * (0.033)
	α-Amylase inhibition (%)	60.651 ± 1.438	59.679 ± 2.785	59.211 ± 2.150	0.336 (0.727)

^1^ The results were expressed as the mean value for the three repeated experiments. ^2^ One-way ANOVA was used, and different letters in the same row (a~b) imply significant differences at * *p* < 0.05, respectively.

**Table 4:** Viscosity of custard according to the concentration of gelling agent.

Concentration (%)	Gelling Agent ^1^			F-Value ^2^ (*p*-Value)
	Locust Bean Gum	κ-Carrageenan	Agar	
0.0	341.667 ± 9.815 ^E^	341.667 ± 9.815 ^E^	341.667 ± 9.815 ^F^	0.000 (1.000)
0.2	606.333 ± 18.583 ^aD^	393.333 ± 11.547 ^cE^	467.000 ± 13.856 ^bE^	157.029 *** (0.000)
0.4	1068.333 ± 31.754 ^aC^	760.333 ± 22.502 ^bD^	584.000 ± 11.269 ^cD^	329.424 *** (0.000)
0.6	1515.000 ± 45.033 ^aB^	1063.667 ± 30.089 ^bC^	819.667 ± 24.542 ^cC^	316.797 *** (0.000)
0.8	1573.333 ± 46.188 ^abB^	1589.667 ± 46.705 ^aB^	1150.667 ± 34.385 ^bB^	9.158 ** (0.003)
1.0	1759.667 ± 52.539 ^bA^	2137.000 ± 64.086 ^aA^	1506.333 ± 39.879 ^cA^	107.175 *** (0.000)
F-value (*p*-value)	705.048 *** (0.000)	1130.355 *** (0.000)	939.407 *** (0.000)	

^1^ The results were expressed as the mean value for the three repeated experiments. ^2^ One-way ANOVA was used, and different letters in the same row (a~c) and column (A~F) imply significant differences at ** *p* < 0.01 and *** *p* < 0.001, respectively.

## Data Availability

Not applicable.
